# Supplementary material for: Targeting RNA:protein interactions with an integrative approach leads to the identification of potent YBX1 inhibitors
Source: eLife. 2023 Jan 18;12:e80387. doi: 10.7554/eLife.80387 (PMC9928419; doi:10.7554/eLife.80387)
Supplement: Figure 2—source data 1. — The scoring function S and the free energy of binding Δ⁢Gbind from ABFE simulations used to identify potential hits are reported, along with the average pocket from NMR and the average from MT bench assay. The structures of the molecules are illustrated in Figure 2. Confirmed hits in vitro and/or in vivo are emphasized using a bold font and a color code: red for hits confirmed in vitro and in vivo, black for in vitro only, green for in vivo only and blue for a negative control that was found potent in vivo but not in vitro. Errors on the computed Δ⁢Gbind range from 0.34 to 0.97 kcal.mol– 1. [file elife-80387-fig2-data1.docx]

**Figure 2-source data 1.** **Classification of the 40 molecules selected using the computational approach.** The scoring function *S* and the free energy of binding ΔG_bind_ from ABFE simulations used to identify potential hits are reported, along with the average pocket <CSP> from NMR and the average <mRNA enrichment slope> for MT bench assay. The structures of the molecules are illustrated in Figure 3 of the main text. Confirmed hits *in vitro* and/or *in vivo* are emphasized using a bold font and a color code: red for hits confirmed *in vitro* and *in vivo*, *black* for *in vitro* only, *green* for *in vivo* only and *blue* for a negative control that was found potent *in vivo* but not *in vitro*. Errors on the computed ΔG_bind_ range from 0.34 to 0.97 kcal.mol^-1^.

|  |  |  |  | *In silico* | | | | *In vitro* | *In vivo* |
| --- | --- | --- | --- | --- | --- | --- | --- | --- | --- |
| Class | Subclass | Label | Traditional Name | Scoring Function *S* | Potential Hit | ΔG_bind_ (kcal.mol^-1^) | Potential Hit | <CSP>pocket (ppm) | <mRNA enrichment slope> |
| *Flavonoids* | *Flavonols* | **F1** | *Quercetin* | 18.05 | YES | -9.68 | **YES** | **0.032** | 0.894 |
|  |  | **F2** | *Quercetagetin* | 21.21 | YES | -7.11 | **YES** | **0.039** | **0.840** |
|  |  | **F3** | *3-O-methylquercetin* | 6.15 | Possible | -10.82 | **YES** | **0.034** | **0.815** |
|  |  | **F4** | *Fisetin* | 11.85 | YES | -6.22 | **YES** | **0.028** | 1.102 |
|  |  | F5 | *Rutin* | -60.14 | NO | -10.08 | NO | 0.009 | 0.955 |
|  |  | F6 | *myricitrin* | -16.63 | NO | -4.86 | NO | **0.021** | 0.870 |
|  |  | **F7** | *Herbacetin* | 37.06 | YES | -9.98 | **YES** | **0.025** | 0.876 |
|  |  | **F8** | *Vincetoxicoside B* | 48.98 | YES | -8.06 | **YES** | **0.016** | 0.859 |
|  | *Flavones* | **F9** | *Scutellarien* | 18.94 | YES | -10.46 | **YES** | **0.020** | 0.998 |
|  |  | F10 | *Luteolin-7-methylether* | 41.75 | YES | -5.19 | YES | - | 0.960 |
|  | *Flavanones* | F11 | *Naringenin* | 17.95 | YES | -5.18 | NO | 0.004 | 0.966 |
|  | *Chalcones* | **C1** | *Butein* | 24.62 | YES | -7.60 | **YES** | **0.026** | **0.801** |
|  |  | **C2** | *Okanin* | 55.28 | YES | -6.96 | **YES** | **0.026** | **0.776** |
|  |  | **C3** | *Robtein* | 34.89 | YES | -7.57 | **YES** | **0.022** | **0.749** |
|  |  | C4 | *Chorilifol B* | 38.87 | YES | -6.02 | YES | - | 0.956 |
|  |  | C5 | *Bavachalcone* | 11.69 | YES | -5.61 | YES | - | 0.812 |
|  |  | **C6** | *homobutein* | -11.02 | NO | -2.23 | NO | 0.004 | **0.826** |
|  |  | C7 | *Cardamonin* | -6.40 | NO | -3.91 | NO | - | 0.981 |
|  |  | **C8** | *-* | 12.14 | YES | -7.34 | **YES** | **0.021** | **0.693** |
|  |  | C9 | *-* | 7.22 | Possible | -5.00 | NO | - | 0.914 |
|  |  | C10 | *-* | -21.68 | NO | -0.98 | NO | - | 0.906 |
|  |  | **C11** | *-* | 8.04 | Possible | -10.08 | **YES** | **0.010** | **0.793** |
|  |  | **C12** | *Lichochalcone B* | 7.77 | Possible | -7.37 | **YES** | **0.013** | **0.786** |
| *Flavonoids Analogs with unknown activity* | | A1 | *-* | -1.87 | Possible | -6.78 | Possible | - | 0.889 |
|  |  | A2 | *-* | -19.51 | NO | -5.90 | NO | - | 1.032 |
|  |  | **A3** | *-* | -59.37 | NO | -17.07 | **Possible** | - | **0.803** |
|  |  | A4 | *-* | -29.79 | NO | -8.73 | NO | - | 0.930 |
|  |  | A5 | *-* | -26.37 | NO | -6.77 | NO | - | 0.983 |
|  |  | A6 | *-* | -4.10 | NO | -4.04 | NO | - | 0.973 |
|  |  | A7 | *-* | -5.35 | NO | -6.31 | NO | - | 0.960 |
|  |  | A8 | *-* | -8.28 | NO | -3.47 | NO | - | 0.885 |
| *FDA approved drugs* | *PARP-1 inhibitors* | **P1** | *Niraparib* | 18.24 | YES | -7.24 | **YES** | **0.034** | **0.732** |
|  |  | P2 | *Olaparib* | -42.50 | NO | -1.66 | NO | 0.007 | 0.906 |
|  |  | P3 | *Talazoparib* | -14.08 | NO | -1.37 | NO | 0.009 | 0.927 |
|  |  | P4 | *Veliparib* | 33.59 | YES | -4.79 | NO | 0.006 | 0.950 |
|  |  | P5 | *Rucaparib* | 17.61 | YES | -5.76 | YES | 0.008 | 0.857 |
|  | *Drugs with cancer or viral indication* | D1 | *Nebivolol* | 19.89 | YES | -12.74 | YES | 0.005 | 1.131 |
|  |  | D2 | *Mefloquine* | 24.44 | YES | -3.49 | NO | 0.006 | 0.967 |
|  |  | D3 | *Icotinib* | 6.14 | Possible | -4.73 | NO | - | 0.975 |
|  |  | D4 | *Cabotegravir* | 12.46 | YES | -13.04 | YES | 0.003 | 0.907 |
